# Traditional Chinese medicine combined with chemotherapy for breast cancer after operation: A systematic review and meta-analysis

**DOI:** 10.1097/MD.0000000000040264

**Published:** 2024-11-15

**Authors:** Yanran Zhang, Lihui Shi

**Affiliations:** a Department of Breast, Tongzhou Maternal & Child Health Hospital of Beijing, Beijing, People’s Republic of China.

**Keywords:** breast cancer, chemotherapy, meta-analysis, postoperative, randomized controlled trial, traditional Chinese medicine

## Abstract

**Background:**

This systematic review aims to explore the effect of traditional Chinese medicine combined with chemotherapy on the clinical efficacy of breast cancer postoperative patients, providing theoretical basis for the treatment of breast cancer postoperative patients with traditional Chinese medicine.

**Methods:**

A comprehensive search was conducted on databases such as China National Knowledge Infrastructure, Wanfang Database, VIP Database, Chinese Biomedical Literature Database, PubMed, and the Cochrane Library from their inception to August 2023 to screen RCTs comparing the effect of traditional Chinese medicine combined with chemotherapy with that of chemotherapy alone on the clinical efficacy of breast cancer postoperative patients. The included literature was systematically reviewed.

**Results:**

A total of 22 RCTs involving 1834 breast cancer postoperative patients were finally included, with 918 patients in the traditional Chinese medicine combined with chemotherapy group (treatment group) and 916 patients in the chemotherapy alone group (control group). Meta-analysis showed that compared with the control group, therapeutical effective rate, CD3, CD4, CD4/CD8, and adverse reactions in the treatment group were all improved, and the incidence of leukopenia and thrombocytopenia after chemotherapy were all reduced, with statistically significant differences.

**Conclusion:**

Traditional Chinese medicine combined with chemotherapy is superior to chemotherapy alone in improving the therapeutical effective rate after breast cancer surgery, reducing chemotherapy adverse reactions, and enhancing immune function.

## 1. Introduction

Breast cancer is currently the most commonly diagnosed type of cancer in women worldwide, and the second leading cause of cancer death in women of all ages and the primary cause of cancer death in women aged 20 to 59.^[1]^ In China, the incidence of breast cancer accounts for 11.19% of the global total, with about 278,900 new cases and 66,000 deaths in 2014, and is growing rapidly.^[2]^

Currently, the treatment for breast cancer is comprehensive and includes surgery, radiotherapy, chemotherapy, molecular targeted therapy, endocrine therapy, traditional Chinese medicine therapy, and other methods. Surgery is the main treatment method for breast cancer, and with the pursuit of beauty by patients, the surgical methods for breast cancer have developed from radical surgery, modified radical surgery, extended radical surgery to breast-conserving surgery, and breast reconstruction surgery.^[3]^ Although surgery is the main treatment for breast cancer, its prognosis is still influenced by various factors, including the pathological type, stage, size, and lymph node metastasis of the tumor. Therefore, finding effective auxiliary treatment methods is crucial for improving the prognosis of breast cancer patients.^[4]^

Chemotherapy is one of the systemic treatments for breast cancer, which can inhibit the proliferation of tumor cells and reduce the recurrence rate of breast cancer after surgery, thereby prolonging the survival of patients. It plays a crucial role in the systemic treatment of breast cancer.^[5]^ However, chemotherapy has numerous side effects, such as suppression of the hematopoietic system, chemotherapy-induced nausea and vomiting, liver and kidney function damage, myocardial injury, and cancer-related fatigue, which can easily hinder the smooth progress of chemotherapy and even affect the chemotherapy effect.^[6]^ With the continuous development of contemporary medicine, traditional Chinese medicine, with its unique advantages, plays a positive role in increasing efficacy, enhancing physical fitness, improving quality of life, and reducing side effects in the comprehensive treatment of tumors.^[7]^ In recent years, more and more studies have begun to explore the effect of traditional Chinese medicine combined with chemotherapy in the treatment of breast cancer. However, due to various reasons, the results of these studies are inconsistent, so a systematic meta-analysis is needed to comprehensively evaluate the efficacy of traditional Chinese medicine combined with chemotherapy in the treatment of breast cancer.

This study aims to systematically conduct a meta-analysis to comprehensively evaluate the efficacy of traditional Chinese medicine combined with chemotherapy in the treatment of breast cancer. We plan to collect and analyze all relevant clinical studies to determine whether traditional Chinese medicine combined with chemotherapy can improve the prognosis of breast cancer patients, as well as its specific efficacy and safety. We hope that this study can provide new ideas and methods for the treatment of breast cancer and provide better treatment options for patients.

## 2. Method

This study was compiled based on the Preferred Reporting Items for Systematic reviews and Meta-Analyses statement.

### 2.1. Databases

Two authors independently finished comprehensive retrieval of related literature in China National Knowledge Infrastructure, Wanfang Database, VIP Database, Chinese Biomedical Literature Database, PubMed, and The Cochrane Library from their start date to August 2023 using computer retrieval. We used the following keywords and medical terms: “Traditional Chinese Medicine,” “Breast Cancer,” “Postoperative,” and “Chemotherapy.”

### 2.2. Inclusion criteria

(1) The patients with breast cancer who have been pathologically diagnosed and undergone surgery, regardless of the type of breast cancer surgery used.
(2) The treatment group received combined traditional Chinese medicine and chemotherapy, while the control group received chemotherapy alone.
(3) Only herbal medicine base on traditional Chinese medicine in treatment group was included.
(4) RCT on the efficacy of combined traditional Chinese medicine and chemotherapy in the treatment of breast cancer after surgery;
(5) The outcome indicators include therapeutical effective rate, adverse reactions, and immune function.

### 2.3. Exclusion criteria

(1) Treatment group did not use traditional Chinese medicine combined with chemotherapy to treat breast cancer after surgery or control group did not use chemotherapy alone.
(2) Treatment group used acupuncture and tuina.
(3) Outcome indicators did not meet the requirements of the study.
(4) Repeated publication.
(5) Animal experiments, mechanism, reviews, protocols, experience, and case reports.
(6) No statistical analysis was conducted in the study.

### 2.4. Data extraction and quality assessment

The quality evaluation tools recommended by the Cochrane 5.1.0 assessment tool to evaluate the methodological quality of the included literature. The evaluation content includes: (1) random sequence generation; (2) allocation concealment; (3) blinding of participants and personnel; (4) blinding of outcome assessment; (5) incomplete outcome data; (6) selective reporting; (7) other bias. The evaluation process was conducted by 2 researchers separately, and any disagreements were decided through discussion.

### 2.5. Data analysis

Used RevMan5.3 provided by the Cochrane Collaboration as the statistical software for meta-analysis. Count data were expressed as relative risk (RR) for effect size, while continuous data were expressed as standardized mean difference for effect size, both with 95% confidence intervals (CI) presented. When the result of the heterogeneity test for included studies was *P* > .01 or *I*^2^ ≤ 50%, a fixed-effects model was used; when the result of the heterogeneity test is *P* ≤ .01 or *I*^2^ > 50%, a random-effects model was used. A *P* value <.05 was considered statistically significant.

## 3. Results

### 3.1. Inclusion criteria and quality assessment of the study

**Figure 1.**
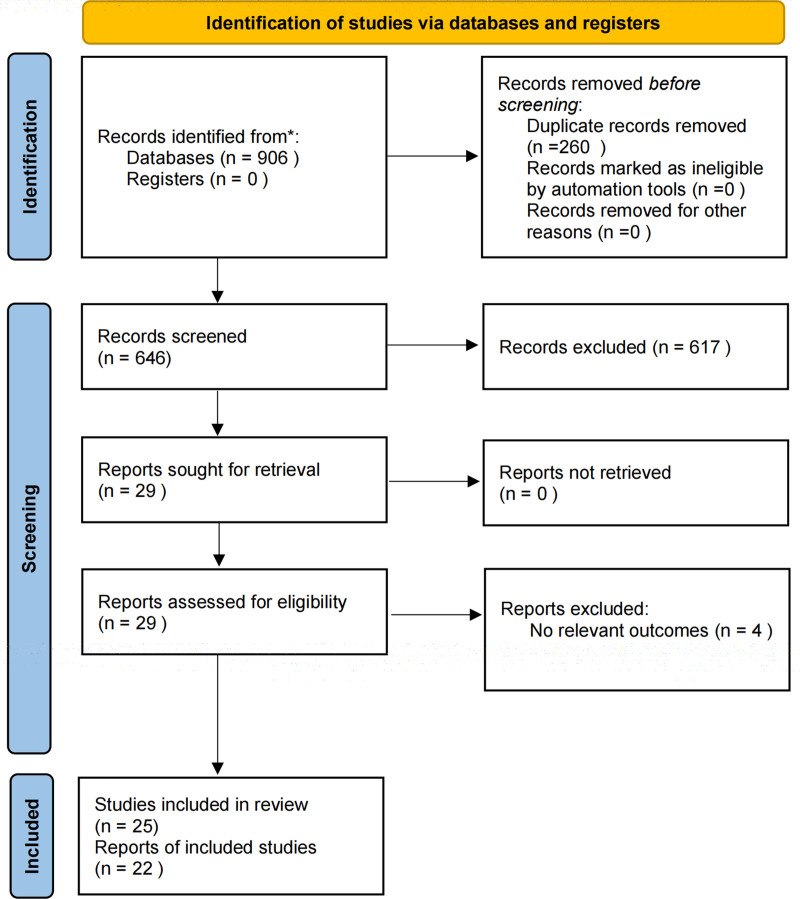
Flow chart of study searching and selection.

**Figure 2.**
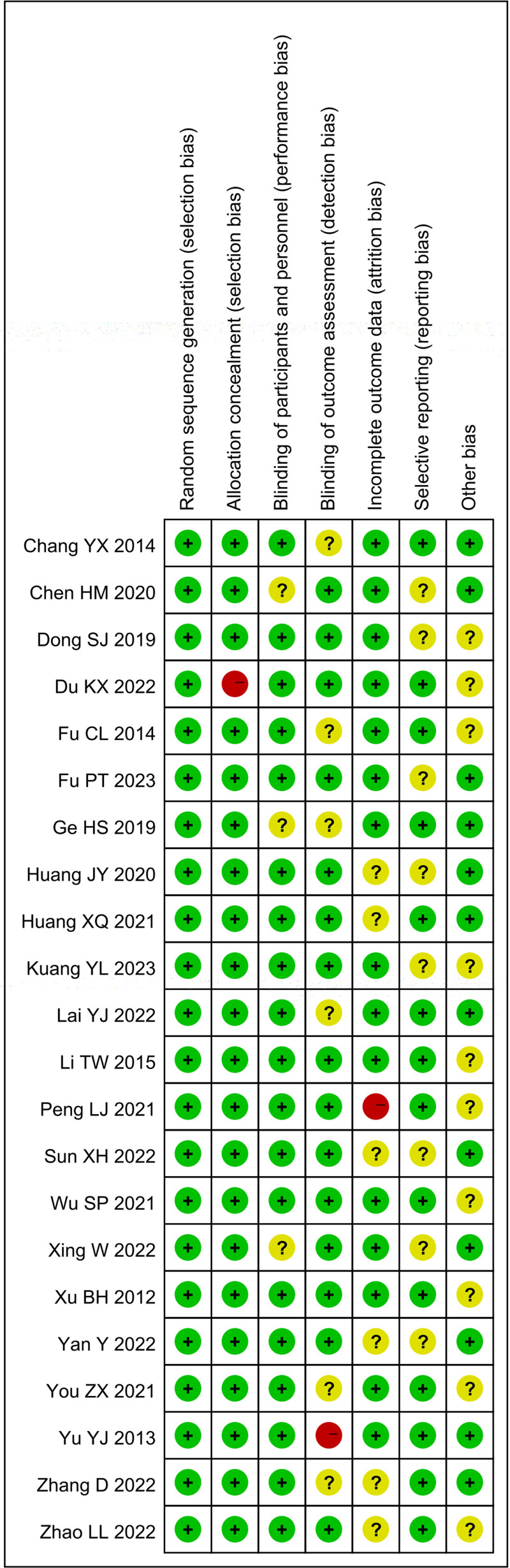
Risk of bias summary.

In Figure 1, according to the inclusion and exclusion criteria, a preliminary search retrieved 906 relevant articles. Using NoteExpress3.2 software, 260 duplicate articles were removed. After reading the titles and abstracts, 617 articles were excluded, leaving 29 articles. After reading the full texts, 7 articles were deleted, and finally 22 articles were included. The basic characteristics and quality evaluation of the included literature are shown in Figure 2 and Table 1. The 22 included articles^[8–29]^ were published between 2012 and 2023. A total of 1834 samples were included, with 918 in the treatment group and 916 in the control group. As shown in Figure 2, all studies mentioned randomization. One of the studies was high risk of bias for the allocation concealment. Three of studies had no description about blinding of participants and personnel. One study had high risk of bias for the incomplete outcome data, whereas 6 studies were unclear. Eight studies were unclear for selective reporting. Ten studies were unclear for other bias.

**Table 1:** Characteristics of included studies.

Included studies	Sample (T/C)	Age (median or mean or range)	TNM stage	Interventions		Outcome indicators
				C	T	
Kuang YL (2023)^[8]^	47/47	T: 53.76 ± 4.17 C: 52.37 ± 3.75	I–III	Dox + CTX (21 days, 4 periods) Doc (d1, 21 days, 4 periods)	Compound Kushen injection (d1–d6) Dox + CTX (21 days, 4 periods) Doc (d1, 21 days, 4 periods)	①②⑤⑥
Fu PT (2023)^[9]^	50/50	T: 47.18 ± 6.40 C: 47.92 ± 6.37	I–III	CTX + Doc + Epir (d1, 21 days, 4 periods)	Fuzheng Xiaoliu Decoction (7 days, 4 periods) CTX + Doc + Epir (d1, 21 days, 4 periods)	①②③④⑤
Chen HM (2020)^[10]^	37/37	T: 45.24 ± 5.41 C: 46.18 ± 5.34	I–IV	CTX + Pir + Fu (28 days, 6 periods)	Erxian Decoction (6 months) CTX + Pir + Fu (28 days, 6 periods)	①③④⑤
Dong SJ (2019)^[11]^	23/22	T: 51.3 ± 7.4 C: 49.7 ± 7.2	I–II	CTX + MTX + Fu (28 days, 1 period)	Fuzheng Quxie Decoction CTX + MTX + Fu (28 days, 1 periods)	③④
Yan Y (2022)^[12]^	40/40	T: 48.62 ± 6.73 C: 49.26 ± 7.49	I–III	CTX + Dox (21 days, 2 periods)	Fuzheng Xiaozheng Decoction (14 days, 2 periods) CTX + Dox (21 days, 2 periods)	②③⑤
Du KX (2022)^[13]^	38/38	T: 47.11 ± 5.25 C: 47.81 ± 4.58	II–III	Dex + Doc (21 days, 4 periods)	Guipi Decoction (21 days, 4 periods) Dex + Doc (21 days, 4 periods)	②③④⑤
Huang JY (2020)^[14]^	41/41	T: 49.3 ± 2.6 C: 48.8 ± 2.5	I–II	Doc + Cap (21days, 6 periods)	Traditional Chinese Medicine (3 months) Doc + Cap (21days, 6 periods)	①⑥
Zhao LL (2022)^[15]^	60/60	T: 46.6 C: 45.8	I–III	Dox + CTX (21 days, 4 periods) Doc (d1, 21 days, 4 periods)	Jianpi Huazhuo Formula (21 days, 8 periods) Dox + CTX (21 days, 4 periods) Doc (d1, 21 days, 4 periods)	②③④⑤
Wu SP (2021)^[16]^	50/50	T: 46.26 ± 3.25 C: 46.23 ± 3.20	II–III	CTX + Dox + Fu (21 days, 4 periods)	Jianpi Xiaoji Decoction (21days, 4 periods) CTX + Dox + Fu (21 days, 4 periods)	③④⑤
Peng LJ (2021)^[17]^	38/38	T: 50.03 ± 7.12 C: 50.25 ± 7.30	II–III	Doc + Epir (21 days, 3 periods)	Qi Shen Decoction (21days, 3 periods) Doc + Epir (21 days, 3 periods)	①③⑤⑥
Xing W (2022)^[18]^	35/35	T: 47.01 ± 6.85 C: 45.65 ± 7.20	I–IV	Doc + Gem (21 days, 6 periods)	Qigui Bawei Decoction (21 days, 6 periods) Doc + Gem (21 days, 6 periods)	③④⑤
You ZX (2021)^[19]^	64/64	T: 43.56 ± 5.23 C: 43.48 ± 5.69	I–III	CTX + MTX + Fu (28 days, 1 period)	Xiaoyao Loubei powder (28 days, 1 period) CTX + MTX + Fu (28 days, 1 period)	①②③⑤
Zhang D (2022)^[20]^	39/39	T: 49.82 ± 8.66 C: 50.15 ± 10.73	I–III	Dox + CTX (21 days, 2 periods)	Yangzheng Mixture (10 days, 2 periods) Dox + CTX (21 days, 2 periods)	①
Sun XH (2022)^[21]^	43/43	T: 53.4 ± 2.8 C: 55.9 ± 2.5	II–III	CTX + Fu + Epir (d1, 21 days, 4 periods)	Yiqi Yangrong Decoction (21 days, 4 periods) CTX + Fu + Epir (d1, 21 days, 4 periods)	①⑥
Huang XQ (2021)^[22]^	40/40	T: 50.12 ± 10.95 C: 51.62 ± 9.82	I–III	AC-T	Qi and nourishing yin therapy AC-T	①⑥
Lai YJ (2022)^[23]^	30/30	T: 41.47 ± 10.34 C: 42.98 ± 11.24	I–III	AC7 (21 days, 7 periods)/AC-T3 ((21 days, 3 periods)	Qi and nourishing yin therapy AC7 (21 days, 7 periods)/AC-T3 ((21 days, 3 periods)	③⑤
Li TW (2015)^[24]^	35/34	T: 47.29 ± 5.62 C: 48.19 ± 5.36	I–IV	CTX + Fu + Epir (d1, d8, 21 days, 4 periods)	Fuzheng Kangai Decoction (21 days, 4 periods) CTX + Fu + Epir (d1, d8, 21 days, 4 periods)	①②③④⑤⑥⑦⑧
Fu CL (2014)^[25]^	30/30	T: 55.47 ± 11.2 C: 54.10 ± 12.2		CTX + ADM + Fu (21 days, 4 periods)	*Solanum nigrum* Decoction (21 days, 4 periods) CTX + ADM + Fu (21 days, 4 periods)	①⑦⑧
Chang YX (2014)^[26]^	30/30	T: 46.5 ± 6.6 C: 47.3 ± 5.7	III–IV	CTX + Fu + Epir (21 days, 4 periods)	Renshen Yangrong Decoction (21 days) CTX + Fu + Epir (21 days, 4 periods)	①⑥⑦⑧
Xu BH (2012)^[27]^	26/26	T: 40. 9 ± 6. 4 C: 41. 2 ± 6. 5	I–III	Pac + Dox (21 days, 3 periods)	Traditional Chinese medicine (21 days, 3 periods) Pac + Dox (21 days, 3 periods)	②③④⑤
Yu YJ (2013)^[28]^	32/32	T: 48.3 ± 3.6 C: 47.9 ± 3.4	I–III	CTX + Fu + Dox (21 days, 3 periods)	Milk Yan Xiao decoction CTX + Fu + Dox (21 days, 3 periods)	①⑥
Ge HS (2019)^[29]^	90/90	T: 43.86 ± 8.19 C: 43.89 ± 8.24	II–III	CTX + Epir + Doc (21 days, 4 periods)	Guyuan Yangxue decoction (7 days, 4 periods) CTX + Epir + Doc (21 days, 4 periods)	①②③④⑤

① Therapeutical effective rate; ② CD3+ proportion; ③ CD4+ proportion; ④ CD8+ proportion; ⑤ CD4+/CD8+ ratio; ⑥ ADRs (adverse reactions); ⑦ reduction in white blood cells; ⑧ reduction in platelets.

ADM = adriamycin, C = control group, Cap = capecitabine, CTX = cyclophosphamide, Dex = dexamethasone, Doc = docetaxel, Dox = doxorubicin, Epir = epirubicin hydrochloride, Fu = fluorouracil, MTX = methotrexate, Pac = paclitaxel, Pir = pirarubicin, T = treatment group.

### 3.2. Therapeutical effective rate

**Figure 3.**
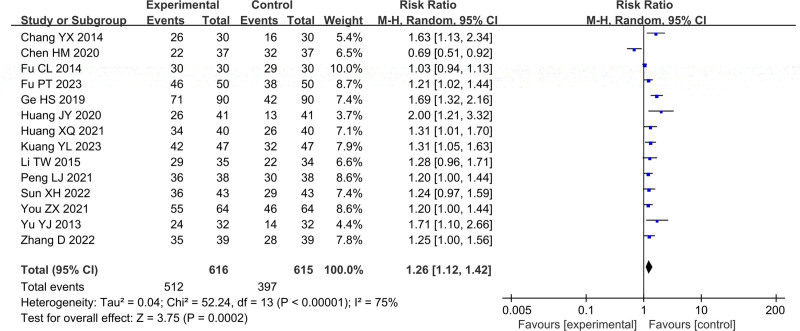
Forest plot of therapeutical effective rate.

In Figure 3, total of 14 studies reported the therapeutical effective rate, including 1231 cases, which 616 cases were in the treatment group and 615 cases were in the control group. After heterogeneity testing, *P* < .00001, *I*^2^ = 75%, random effects model was used. The meta-analysis results showed that therapeutical effective rate of patients in the treatment group was higher than that of the control group (RR = 1.26, 95%CI [1.12, 1.42], *P* = .0002).

### 3.3. Immune function

**Figure 4.**
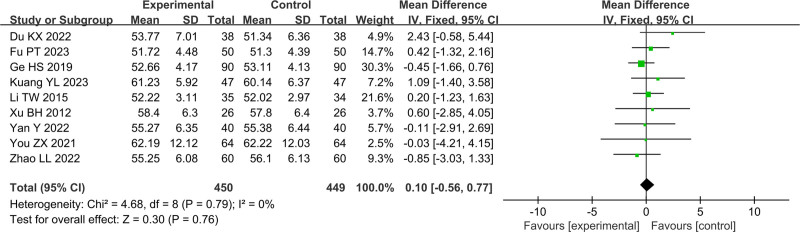
Forest plot of CD3^+^ before treatment.

**Figure 5.**
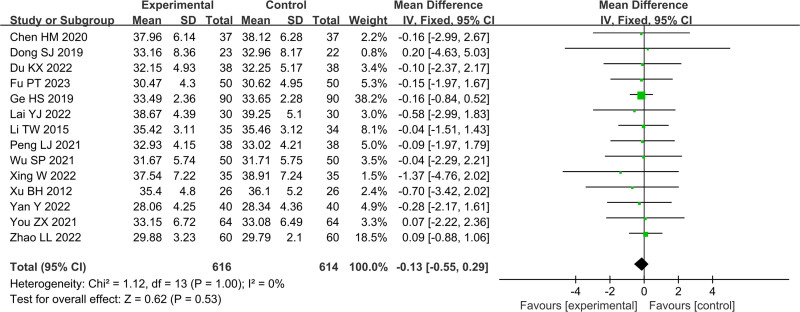
Forest plot of CD4^+^ before treatment.

**Figure 6.**
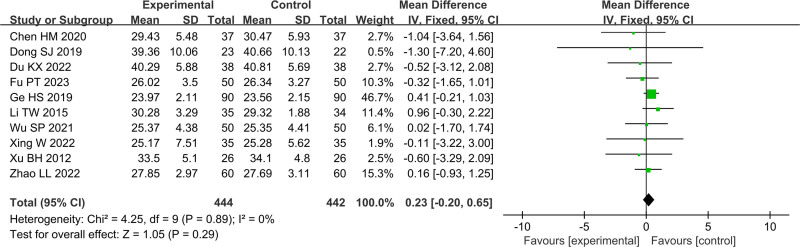
Forest plot of CD8^+^ before treatment.

**Figure 7.**
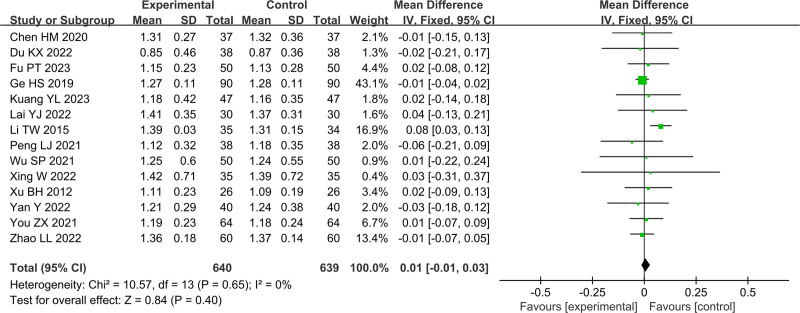
Forest plot of CD4^+^/CD8^+^ before treatment.

**Figure 8.**
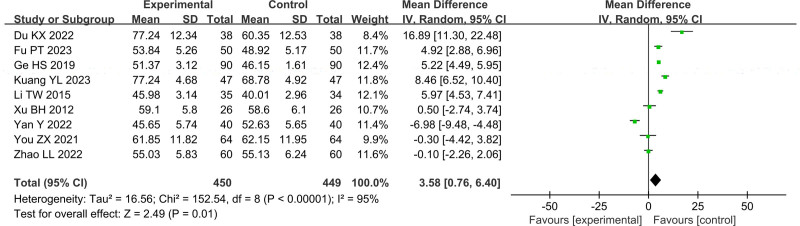
Forest plot of CD3^+^ after treatment

**Figure 9.**
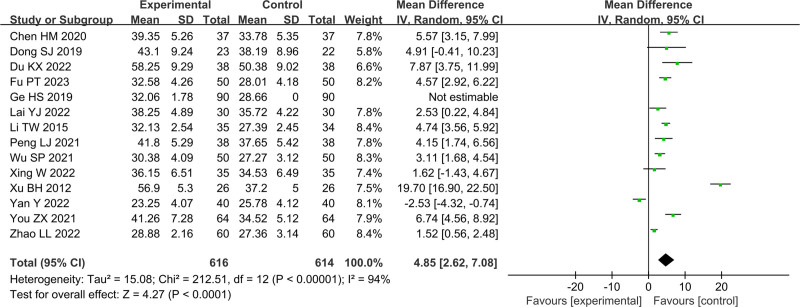
Forest plot of CD4^+^ after treatment.

**Figure 10.**
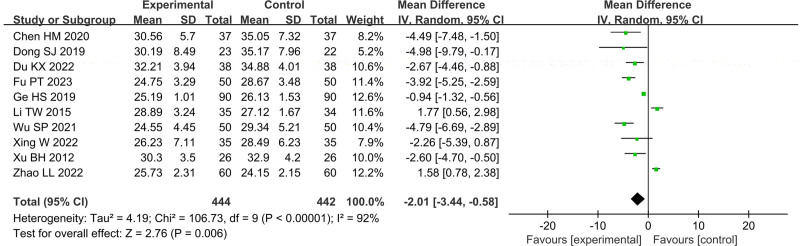
Forest plot of CD8^+^ after treatment.

**Figure 11.**
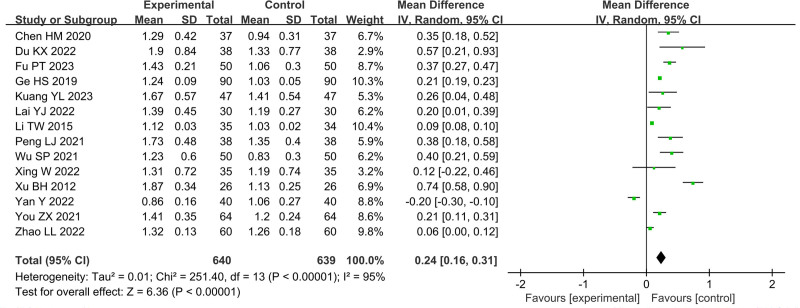
Forest plot of CD4^+^/CD8^+^ after treatment.

In Figures 4–11, 9 studies including 889 cases reported CD3^+^, 14 studies including 1230 cases reported CD4^+^, 10 studies including 886 cases reported CD8^+^, 14 studies including 1279 cases reported CD4^+^/ CD8^+^. In Figures 4–7, after heterogeneity testing, the *P* values of CD3^+^, CD4^+^, CD8^+^, and CD4^+^/ CD8^+^ were 0.79, 1.00, 0.89, 0.65, and the *I*^2^ values of all were 0%. A fixed effect model was used. The meta-analysis results showed that the differences in pretreatment T lymphocytes (CD3^+^, CD4^+^, CD8^+^, and CD4^+^/CD8^+^) cell levels between the 2 arms were not significant (CD3^+^, MD = 0.10, 95%CI [‐0.56, 0.77], *P* = .0.76; CD4^+^, MD = ‐0.13, 95%CI [‐0.55, 0.29], *P* = .53; CD8^+^, MD = 0.23, 95%CI [‐0.20, 0.65], *P* = .0.29; CD4^+^/CD8^+^, MD = 0.01, 95%CI [‐0.01, 0.03], *P* = .40). After treatment, the *I*^2^ values of CD3^+^, CD4^+^, CD8^+^, and CD4^+^/ CD8^+^ were 95%, 94%, 92%, 95%, and the *P* < .00001. A random effects model was used. The meta-analysis results showed that immune function of patients in the treatment group was better than that of the control group (CD3^+^, RR = 3.58, 95%CI [0.76, 6.40], *P* = .01; CD4^+^, RR = 4.85, 95%CI [2.62, 7.08], *P* < .0001; CD8^+^, RR = ‐2.01, 95%CI [‐3.44, ‐0.58], *P* = .006; CD4^+^/CD8^+^, RR = 0.24, 95%CI [0.16, 0.31], *P* < .00001).

### 3.4. Adverse reactions (ADRs)

**Figure 12.**
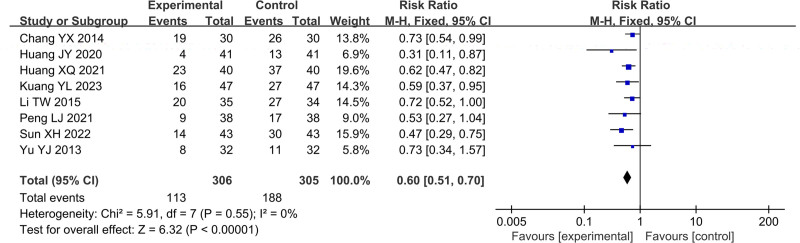
Forest plot of ADRs. ADRs = adverse reactions.

In Figure 12, total of 8 studies reported the ADRs, including 811 cases, which 306 cases were in the treatment group and 305 cases were in the control group. After heterogeneity testing, *P* = .55, *I*^2^ = 0%, random effects model was used. The meta-analysis results showed that therapeutical effective rate of patients in the treatment group was higher than that of the control group (RR = 0.60, 95%CI [0.51, 0.70], *P* < .00001).

### 3.5. Platelet

**Figure 13.**
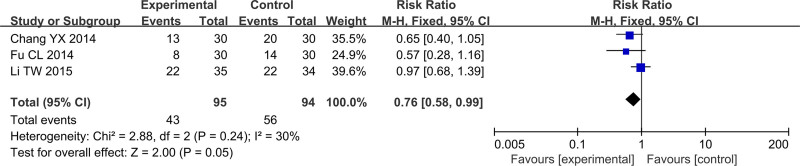
Forest plot of PLT. PLT = platelet.

In Figure 13, total of 3 studies reported the platelet (PLT), including 189 cases, which 95 cases were in the treatment group and 94 cases were in the control group. After heterogeneity testing, *P* = .24, *I*^2^ = 30%, random effects model was used. The meta-analysis results showed that PLT of patients in the treatment group was higher than that of the control group (RR = 0.76, 95%CI [0.58, 0.99], *P* = .05).

### 3.6. White blood cell (WBC)

**Figure 14.**
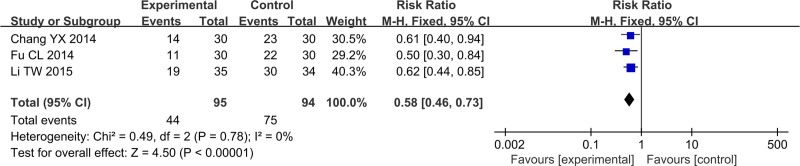
Forest plot of WBC. WBC = White blood cell.

In Figure 14, total of 3 studies reported the WBC, including 189 cases, which 95 cases were in the treatment group and 94 cases were in the control group. After heterogeneity testing, *P* = .78, *I*^2^ = 0%, random effects model was used. The meta-analysis results showed that PLT of patients in the treatment group was higher than that of the control group (RR = 0.58, 95%CI [0.46, 0.78], *P* < .00001).

### 3.7. Publication bias

**Figure 15.**
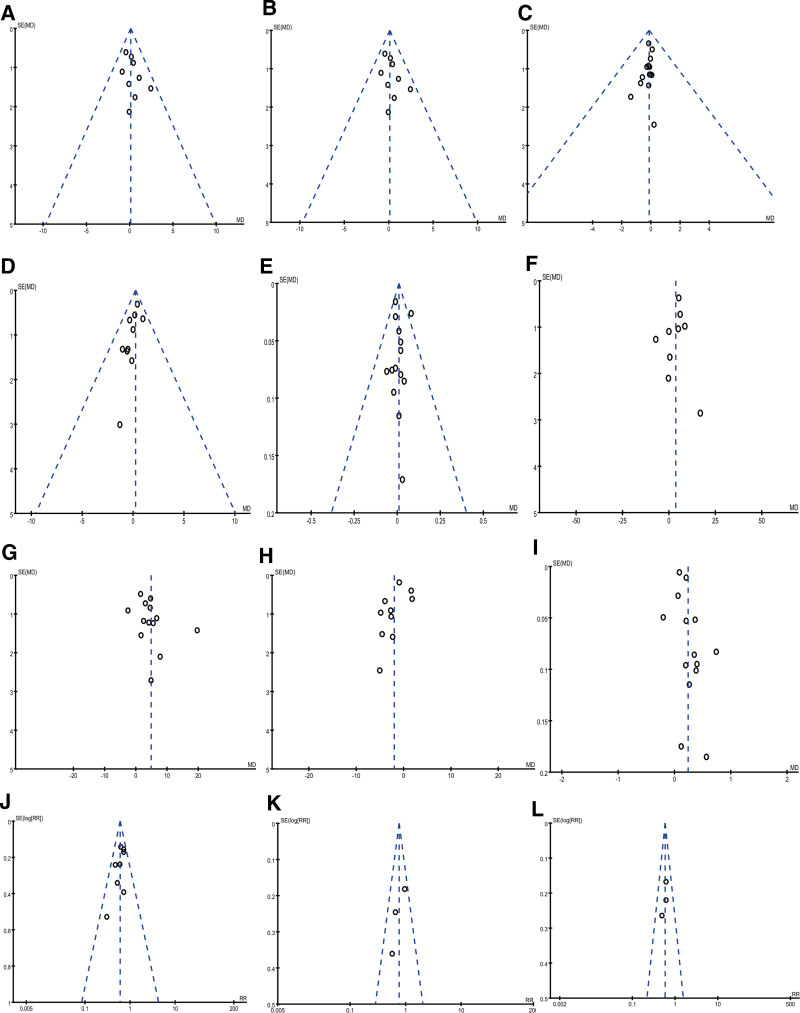
Funnel plot. (A) Therapeutical effective rate; (B) CD3^+^ before treatment; (C) CD4^+^ before treatment; (D) CD8^+^ before treatment; (E) CD4^+^/CD8^+^ before treatment; (F) CD3^+^ after treatment; (G) CD4^+^ after treatment; (H) CD8^+^ after treatment; (I) CD4^+^/ CD8^+^ after treatment; (J) ADRs; (K) PLT; (L) WBC. ADRs = adverse reactions, PLT = platelet, WBC = white blood cell.

Figure 15 reported the funnel plot of therapeutical effective rate (A); CD3^+^ before treatment (B); CD4^+^ before treatment (C); CD8^+^ before treatment (D); CD4^+^/CD8^+^ before treatment (E); CD3^+^ after treatment (F); CD4^+^ after treatment (G); CD8^+^ after treatment (H); CD4^+^/CD8^+^ after treatment (I); ADRs (J); PLT (K); WBC (L). Each plot was symmetric, indicating no publication bias.

## 4. Discussion

Surgery is an indispensable and important part of comprehensive treatment for breast cancer and has become the preferred method for treating breast cancer. Postoperative chemotherapy helps to kill residual tumor cells in the body, improve surgical efficacy, and control the primary lesion.^[5]^ However, both surgery and chemotherapy belong to the methods of eliminating evil, and eliminating evil is easy to damage the vital energy. From the perspective of traditional Chinese medicine, these treatment methods all have the problem of eliminating evil too much, causing the vital energy of patients to be more deficient. The key to treating masses lies in knowing when to attack and when to supplement. Therefore, patients who receive adjuvant chemotherapy after breast cancer surgery should be accompanied by traditional Chinese medicine treatment to achieve the effect of tonifying the vital energy and eliminating evil.^[11]^

Postoperative chemotherapy can control the disease condition in a short period of time, but a large amount of evidence shows that it has varying degrees of toxic side effects, which are mainly due to the damage to normal cells while killing cancer cells. Although current drug development has gradually introduced drug target selection to minimize damage to normal cells, side effects such as hair loss, nausea, and bone marrow suppression still occur during chemotherapy, which reduces the quality of life for patients. Traditional Chinese medicine treatment can assist in treating the adverse reactions caused by chemotherapy. For example, a meta-analysis by Pan et al^[30]^ showed that modified Xiaoyao san combined with chemotherapy has significant advantages over chemotherapy alone in improving quality of life, reducing clinical symptoms, reducing nausea, vomiting, and cardiac toxicity, and prolonging survival time in the treatment of breast cancer. This is consistent with the results obtained in this study, proving the significant advantages of traditional Chinese medicine combined with chemotherapy in the treatment of breast cancer. This study demonstrates that traditional Chinese medicine combined with chemotherapy is superior to chemotherapy alone in improving the effectiveness of postoperative treatment for breast cancer, reducing adverse reactions, and enhancing immune function.

The studies included in this research were all from China and their quality was generally low, which directly affected the quality of the research results. The herbal medicines used in the included studies were also different, which may lead to significant differences between different studies. In addition, the degree of detail in the baseline data, the inconsistent clinical staging of tumors, the different chemotherapy regimens, the unclear TCM syndromes, the different treatment durations, and various potential confounding factors in the experiment all became potential heterogeneity. In order to more comprehensively and objectively evaluate the effectiveness and practicality of the combination of TCM and chemotherapy in the treatment of breast cancer after surgery, we look forward to more RCTs with large samples and multicenter studies in the future, providing more scientific and rigorous evidence for clinical practice. In future further studies, we hope that other regions will also involve in the TCM field and provide more high-quality research literature. More articles can unify the baseline data, exclude different chemotherapy drugs, different TCM syndromes, and different treatment durations as influencing factors, and then make corresponding analyses to draw more authentic and reliable conclusions.

The results of this study showed that compared with the control group, therapeutical effective rate, CD3, CD4, CD4/CD8, and ADRs in the treatment group were all improved, and the incidence of leukopenia and thrombocytopenia after chemotherapy were all reduced, with statistically significant differences. Traditional Chinese medicine combined with chemotherapy is superior to chemotherapy alone in improving the therapeutical effective rate after breast cancer surgery, reducing chemotherapy adverse reactions, and enhancing immune function.

## Author contributions

**Data curation:** Lihui Shi.

**Writing – review & editing:** Yanran Zhang, Lihui Shi.
